# Interactive effects of high planting density and drought on physiological traits and yield in tomato

**DOI:** 10.1002/jsfa.70368

**Published:** 2025-12-03

**Authors:** Silvana Francesca, Valerio Cirillo, Alessia Cuccurullo, Rana Choukri, Nausica Pollaro, Matteo Addonizio, Mohamed Faize, Mourad Baghour, Maria Manuela Rigano

**Affiliations:** ^1^ Department of Agricultural Sciences University of Naples “Federico II” Portici Italy; ^2^ Laboratory OLMAN‐BPGE, Multidisciplinary Faculty of Nador Mohamed First University – Oujda Nador Morocco; ^3^ Laboratory of Plant Biotechnology, Ecology and Ecosystem Valorization, CNRST URL10, Faculty of Sciences University Chouaib Doukkali El Jadida Morocco

**Keywords:** tomato, proximity shade, yield, fruit quality, drought stress

## Abstract

**Background:**

This study investigated the combined effects of high planting density and drought stress on morpho‐physiological traits, yield, and fruit quality in tomato plants. The research addresses knowledge gaps in plant responses to multiple concurrent stressors and explores strategies for maximizing yield per cultivation area under water scarcity conditions. Two experimental approaches were employed: a controlled environment study with 30‐day‐old tomato seedlings grown under low density (LD) or high density (HD) conditions with normal or restricted water supply, and a field experiment carried out up to the fruiting stage. Measurements included morphological traits, photosynthetic parameters, oxidative stress markers, pigment content, gene expression of shade marker genes, yield components, and fruit quality attributes.

**Results:**

HD cultivation triggered typical shade avoidance syndrome responses, but unexpectedly enhanced photosynthetic rates compared to LD. HD did not exacerbate the physiological response to drought but did reduce per‐plant yield when combined with drought. Light quality modifications under HD led to increased lycopene content in fruits, suggesting potential nutritional quality benefits.

**Conclusions:**

The findings challenge simplistic views of combined stress effects, revealing that moderate shade from HD cultivation may mitigate certain aspects of drought stress. While combined HD and drought reduced yield, the enhanced photosynthetic efficiency and improved fruit quality parameters suggest optimized HD cultivation could represent a viable strategy for sustainable intensification of tomato production under water‐limited environments. © 2025 The Author(s). *Journal of the Science of Food and Agriculture* published by John Wiley & Sons Ltd on behalf of Society of Chemical Industry.

## INTRODUCTION

In the present era, the increasing demand for food and the continuous deterioration of limited available arable land necessitates growing more resilient crops and increasing plant productivity.^1^ For these reasons, farming practices are shifting towards cultivating plants at higher densities to increase yield per unit of cultivation area. As an example, for tomato (*Solanum lycopersicum*), a crop known for its global importance, the recommended planting densities average 3.6 plants per square meter for plants with indeterminate growth habits growing under a glasshouse and 1.1 plants per square meter for open‐field plants with a determinate growth habit.^2^ High density (HD) planting during tomato cultivation can be a valid alternative strategy to maximize yield per land area^3^; however, it can also cause decreases in the biomass and in the yield per plant and affect the efficiency of resources, as plants actively compete for water and essential nutrients.^2^ Indeed, when the density exceeded the optimal range, HD planting was demonstrated to hamper fruit set and reduce fruit size and number in tomato.^4^ Close vegetation proximity associated to HD planting may change the quality (Red/Far‐red (R/FR) ratio) and intensity of the light^5^ and this could lead to a suboptimal photosynthetic rate.^3^, ^6^ Plants under HD may also react to the natural proximity shade provided by neighboring plants through the shade avoidance syndrome (SAS), a series of responses triggered by a low R/FR ratio perceived by phytochromes, as also reported by Burbano *et al*.^7^ SAS can cause various developmental changes including hypocotyl and stem elongation, apical dominance, and alteration of flowering time.^2^, ^8^ However, exposure to low R/FR light ratio in tomato was able to inhibit fruit weight loss and increase the content of lycopene and β‐carotene in tomato.^9^ That said, the effect of HD planting on fruit quality has still not been fully analyzed. Variations in light quality and quantity caused by plant–plant shading can also trigger several hormonal changes.^5^ Interestingly, numerous studies revealed that shading stress greatly affects the activity of phytohormones and may increase abscisic acid (ABA) content. Asghar *et al*.^10^ demonstrated that pretreatment of shade caused increases in ABA contents and up‐regulation of ABA biosynthetic and signaling genes enhancing drought tolerance in soybean seedlings. Analogously, shade treatments were shown to increase ABA levels in tomato leaves and Arabidopsis buds and leaves, with effects on the hyponastic response and bud growth.^11^ That said, the relationship and mechanisms of the interactive effects of the two stress factors, shade and drought, are not entirely known,^10^ and this is particularly important considering that with higher planting density also comes the challenge of water deficit.^1^ One hypothesis is that shade may increase the effect of drought since plants may need to allocate more resources to the shoot under shade, reducing water absorption.^1^ Another hypothesis predicts that shade might reduce air and leaf temperature, leaf vapor‐pressure deficit and oxidative stress, allowing better tolerance to drought.^1^ Considering the worldwide scarcity of agricultural lands, understanding plants response to combined shade and drought is pivotal to define a strategy to increase crop productivity per square meter without incurring into competition for the water resource. Here, we first analyzed the morphological, physiological, and biochemical mechanisms activated in tomato plants under co‐occurring HD and drought under controlled conditions. Then, taking into consideration that crops are often subjected to co‐occurring shade and water scarcity in the field, another experiment was conducted to investigate the effect of these two stresses in an open field trial. Altogether, the different trials here reported allowed us to deeply analyze the relationship between shade and drought and their interactive effects on crop productivity and fruit quality.

## MATERIALS AND METHODS

### Experimental design and growth conditions in a controlled growth chamber

Seeds of *S. lycopersicum* cv. MoneyMaker were sown in styrofoam trays and then, 1 month after germination, transplanted in plastic pots (11 cm diameter, 9 cm height) with commercial substrate in one controlled chamber located at the Department of Agricultural Science, University of Naples (Naples, Italy). The climate settings of the chamber were 24 ± 3 °C during the day and 18 ± 3 °C during the night.^7^ Plants were grown under a photoperiod of 16 h, from 06:00 to 22:00, provided by light‐emitting diodes (LEDs, C‐led; Cefla Company, Imola (BO), Italy). Plants were watered by drip irrigation, with one 4 L per hour emitter per pot. Light measurements of photosynthetic active radiation (PAR) and light quality were recorded using a handheld spectral radiometer (MSC15; Gigahertz‐Optik, Turkenfeld, Germany). The density factor was imposed by growing plants at 69 and 16 m^−2^ for HD and the low density (LD) treatment, respectively, achieved by using 12 cm × 12 cm spacing between plants and rows for HD and 25 cm × 25 cm spacing between plants and rows for LD. Drought stress was imposed on plants growing under HD or LD by water withholding and started 12 days after transplant, when plants showed morphological traits linked with the shade avoidance responses (stem elongation and lower leaf mass per area (LMA)). Irrigation was interrupted by removing the drip emitter from the pot until the plants showed significant phenotypical changes. This experiment was replicated under similar condition at the Nador Multidisciplinary Faculty (Morocco); however, the measurements of light intensity and quality were not performed, and plants were not subjected to drought. All the results of this experiment have been reported as Supporting Information. In Italy and in Morocco ten plants per treatment were tested.

### Experimental design and growth conditions in the open field trial

The open field experiment was conducted in the experimental farm of the University of Naples ‘Torre Lama’ located in Bellizzi, Salerno, Italy (latitude 40°31′ N; longitude 14°58′ E) on a clay‐loam soil to assess the response of tomato yield components to HD planting (HD cultivation) as compared to normal farmer practice (LD cultivation). Four weeks after sowing, at the third true leaf fully expanded, tomato plants (cv. MoneyMaker) were transplanted in an open field. The distance plant to plant in LD and HD were 33 and 16 cm, respectively. Plants were arranged in a randomized complete block design with factorial arrangement using six replicates per treatment and six plants per biological replication. The experimental field was irrigated based on the evapotraspiration demand by drip irrigation system with 4 L per hour emitter per plant. To give a uniform amount of water, the HD lines were provided with two irrigation pipes. Drought stress was imposed at the flowering and fruit development stage, from first fruit set to first fruit maturity, by suspending irrigation for two irrigation turns.

### Plant growth measurements and final harvest

At the end of the experiment in controlled chambers, the height of the plant, internode length and the diameter of the stem of six plants for each treatment were measured with a meter and a digital caliper (Kynup; Shenzhen, China). For the LMA determination, one leaf was detached, scanned for leaf area measurement and dried at 60 °C until constant weight. LMA was calculated as the ratio between leaf dry weight and leaf area. Tomato plants were uprooted and the shoots harvested were then dried to constant weight in an oven at 80 °C for 72 h for dry biomass (dry weight, DW). In the open field trial marketable fruit yield (considering only the fresh weight (FW) of red ripe fruit) was recorded. Titratable acidity and firmness were evaluated on fresh fruit collected at the red ripe stage. The determination of pH was carried out by using a pH meter (Mettler‐Toledo; Milan, Italy), and the total acidity was determined by titrating 10 mL of tomato juice with a solution of 0.1 mol L^−1^ sodium hydroxide. Fruit firmness, expressed as the maximum force (in kg cm^−2^) needed for penetration of the probe (8 mm diameter) into the fruit through the tomato skin, was measured using a penetrometer (PCE‐PTR200 penetrometer; Capannori, Italy). Ten fruits at red ripe stage from each replicate and for each parameter were harvested to perform qualitative analysis.

### Gene expression analyses

The expression of shade markers genes in leaf samples from plants grown under HD or LD in controlled chambers was verified by real‐time polymerase chain reaction (RT‐PCR) amplification. Total RNA was isolated from tomato leaves by using the ISOLATE II RNA Plant Kit (Meridian Bioscience; Cincinnati, OH, USA) according to the method reported by the manufacturer. Total RNA (1 μg) was treated by the Tetro complementary DNA (cDNA) Synthesis Kit (Meridian Bioscience; Cincinnati, OH, USA). For each RT‐PCR reaction, 1 μL of cDNA was mixed with 6.25 μL SYBR Green PCR master mix (Applied Biosystems, Foster City, CA, USA) and 1.25 μL each of forward and reverse primers (Table S1) in a final volume of 13 μL. The reaction was carried out by using the 7900HT Fast‐Real Time PCR System (Applied Biosystems, Foster City, CA, USA). All reactions were run in triplicate for each of the three biological replicates and a housekeeping gene coding for the elongation factor 1‐alpha (*Ef1‐α* – *Solyc06g005060*) was used as reference gene.^12^ Low density treatment was selected as calibrator. Quantitative results were expressed as the mean value ± standard error.

### Leaf gas exchange

Leaf gas exchange was performed by using the Li6800 portable photosynthesis system (LiCor, Lincoln, NE, USA). Measurements were carried out in the morning (09:00–11:30 h) on fully expanded mature leaf per plant with the following environmental parameters: photosynthetic photon flux (PPF) density of 1200 μmol photons m^−2^ s^−1^, ambient μmol CO_2_ mol^−1^, relative humidity 50–55%, and fixed temperature regimen at 25 °C (considered as ambient control). Carbon dioxide (CO_2_) assimilation (μmol CO_2_ m^−2^ s^−1^, *A*_n_), stomatal conductance (mol H_2_O m^−2^ s^−1^, *g*_s_) and transpiration rate (mmol H_2_O m^−2^ s^−1^, *E*) were measured. All the measurements were determined on at least six well‐exposed and fully expanded leaves per treatment.

### Content of photosynthetic pigments and lipophilic compounds

The evaluation of total carotenoids and chlorophylls was carried out according to Rigano *et al*.^13^ on leaf materials from plants grown in controlled chambers. To obtain the lipophilic extract, 0.30 g sample were extracted with 24 mL acetone/hexane (40:60, *v/v*). The mixture was centrifuged at 20 000 × *g* for 5 min at 4 °C. For carotenoids and chlorophylls a and b determination, absorbance of lipophilic extracts was read at 470, 663 and 645 nm, respectively. All results were converted into mg 100 g^−1^ FW. Lipophilic content analyses were also carried out on tomato fruit at the red ripe stage from plants grown in the open field. The same protocol as described before was used starting from 0.500 mg of tomato berries ground with liquid nitrogen. For lycopene and β‐carotene levels, absorbance was read at 505 and 453 nm, respectively. All results were converted into mg 100 g^−1^ FW. Three separated biological replicates for each sample and three technical assays for each biological repetition were measured.

### Hydrogen peroxide, malondialdehyde and proline determination

Quantification of hydrogen peroxide (H_2_O_2_) and malondialdehyde (MDA) content was carried out on leaf material using a colorimetric method as reported in Francesca *et al*.^14^ The concentration of H_2_O_2_ was expressed in mmol g^−1^ FW. For the MDA content the concentration was expressed as quantity of MDA–thiobarbituric acid complex. Proline was determined according to Carillo and Gibon.^15^ Proline was extracted using a cold extraction procedure by mixing 20–50 mg FW aliquots with 0.4–1 mL of ethanol/water (40:60 *v/v*). The resulting mixture was left overnight a 4 °C and then centrifuged at 14 000 × *g* (5 min). A 0.5 mL aliquot of extract was added to 1 mL of a 1% (*w/v*) solution of ninhydrin in 60% (*v/v*) acetic acid and ethanol 20% (*v/v*). After homogenization the mix was heated at 95 °C for 20 min. After centrifugation, the absorbance was read at 520 nm. For all different analyses six biological replicates per density and water treatment were used. Three technical assays per replicate were analyzed.

### Statistical analysis

Quantitative parameters were expressed as the mean value ± standard error. Differences among controlled and treated samples were obtained using R programming language (https://www.R-project.org/). Two‐way analysis of variance (ANOVA) was adopted to compute ANOVA. To compare means within each parameter, Turkey *post hoc* test was performed. Differences of *P* < 0.05 were considered significant. Moreover, the significant differences among treatments were estimated using Student's *t*‐test.

## RESULTS AND DISCUSSION

### High density cultivation activates shade avoidance syndrome

**Figure 1:**
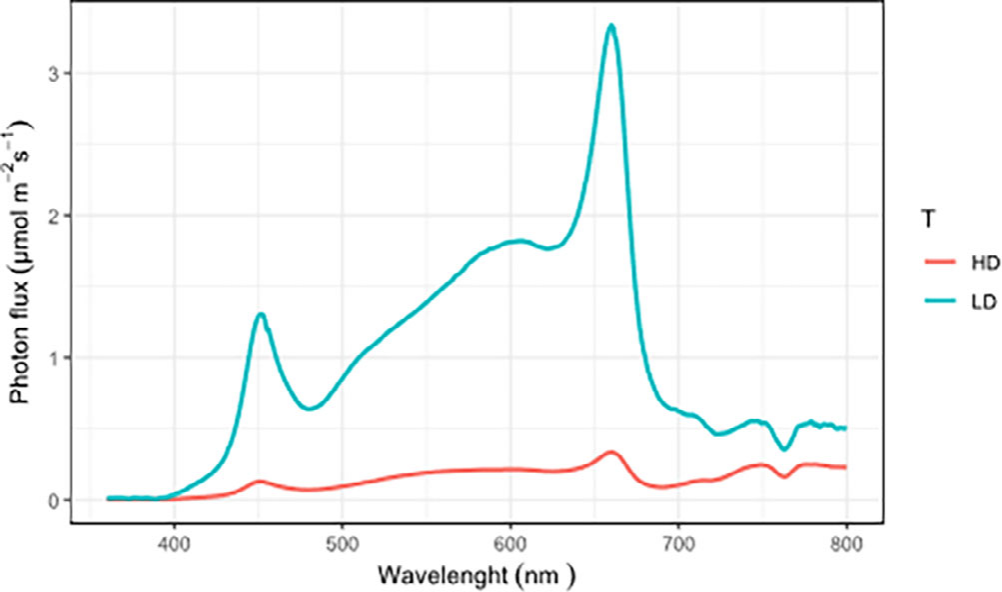
Spectral distribution of the light under low density (LD) and high density (HD) treatment.

**Figure 2:**
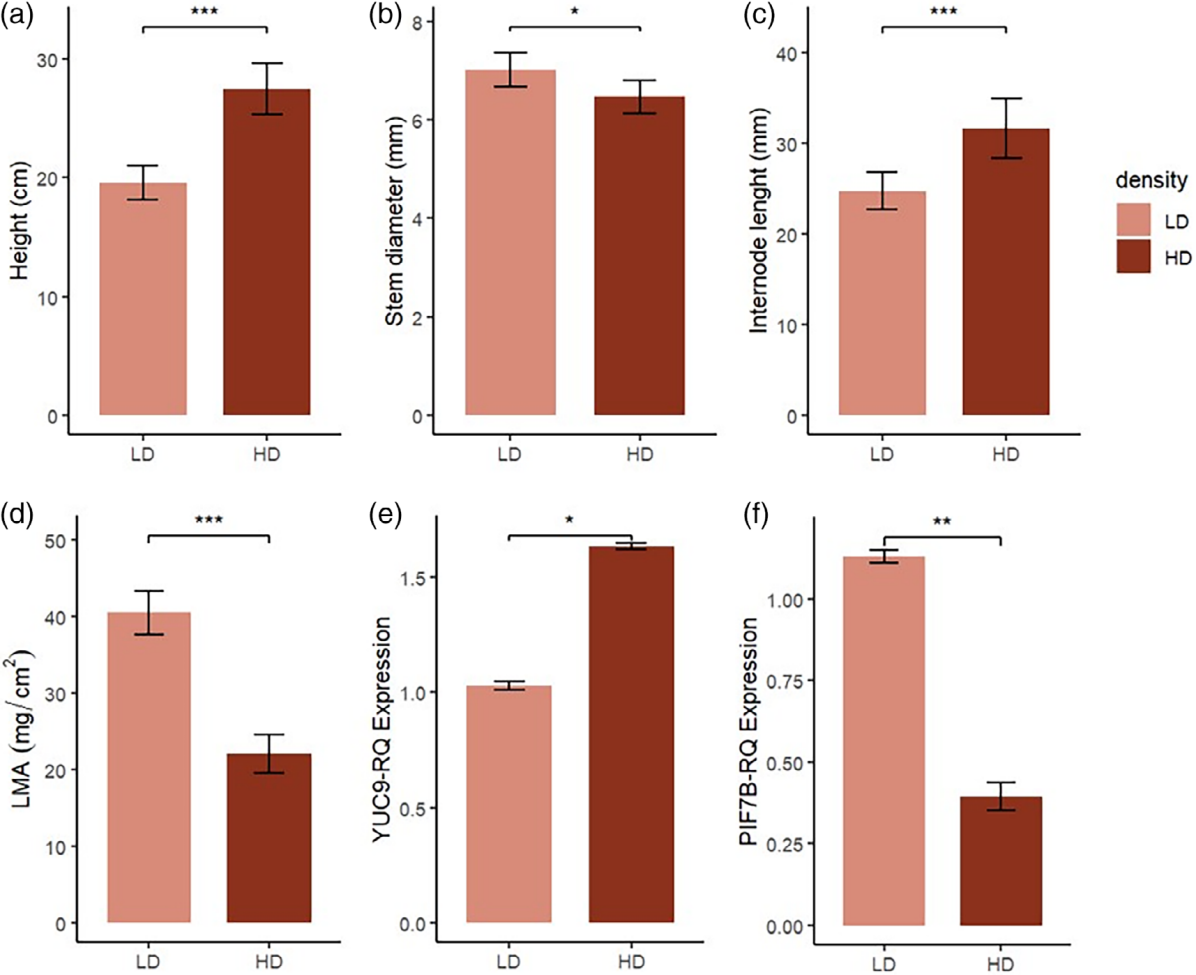
Morphological parameters and gene expression profiles of selected shade markers genes measured in tomato plants under low density (LD) or high density (HD): (a) plant height; (b) stem diameter; (c) internode length; (d) leaf mass per area (LMA); e) relative expression of *YUC9* gene and (f) relative expression of *PIF7b* gene. Data are represented as the mean ± standard error. Asterisks indicate significant differences according to Student's *t*‐test (*P < 0.05).

In plants HD cultivation may increase competition for light that is exacerbated by shade characterized by low R/FR light ratio and a reduction of light intensity. In this article we first investigated if plants react to these light conditions through SAS, a series of responses normally activated by low R/FR light ratio and perceived by phytochromes. Initially, spectral light quality and distribution were recorded for tomato plants grown under LD and HD conditions (Table 1 and Fig. 1). Results from light distribution analyses showed that the amount of PAR intercepted by tomato plants was deeply affected by plant density. Specifically, the HD treatment had a PAR of 64.30 μmol m^2^ s^−1^ compared to 436.08 μmol m^2^ s^−1^ for LD (Table 1). Plant density had also significant effects on the R/FR light ratio; particularly, we noticed a lower R/FR ratio under HD compared to LD. A drastic decrease in the amount of light absorbed at 700–800 nm was observed (Table 1). This setup led to drastic differences in the R/FR fractions. The red fraction of the LD was 42% (183 mol m^−2^ s^−1^) over PPF (436 mol m^−2^ s^−1^), while the red fraction of the HD was 29% (20 mol m^−2^ s^−1^) (Table 1). On the contrary, the percentage of the FR fraction showed an opposite trend, being higher in the HD treatment (35%) compared to the LD treatment (12%) (Table 1). Here we demonstrated that these altered light conditions triggered classic SAS responses in tomato plants. Indeed, plants under HD exhibited increased height (28.3 cm *versus* 19.5 cm in LD, Fig. 2(a)) and internode length (32 cm *versus* 25 cm in LD, Fig. 2(c)), reduced stem diameter (6.4 mm *versus* 7.0 mm in LD, Fig. 2(b)), and lower LMA (22.1 mg cm^−2^ *versus* 40.2 mg cm^−2^ in LD; Fig. 2(d)). Using the same HD set‐up and the same genotype, similar results were obtained in another parallel experiment carried out in Morocco (Table S2), further confirming the effect of HD and LD on morphological traits in tomato. These morphological adaptations align with well‐established shade avoidance strategies aimed at repositioning photosynthetic tissues in more favorable light conditions.^7^, ^16^

**Table 1:** Summary of the spectral qualities tested for the low density (LD) and the high density (HD) treatment

	Low density	High density
Photon flux (μmol m^−2^ s^−1^)		
UV‐A (360–400 nm)	0.59 ± 0.07 (0.14%)	0.12 ± 0.01 (0.19%)
Blue (400–500 nm)	61.43 ± 4.54 (14.09%)	6.60 ± 0.69 (9.49%)
Green (500–600 nm)	139.56 ± 9.23 (32.02%)	17.69 ± 2.76 (26.79%)
Red (600–700 nm)	183.46 ± 11.06 (42.11%)	20.15 ± 3.12 (28.57%)
Far‐red (700–800 nm)	51.03 ± 8.41 (11.64%)	19.74 ± 1.78 (34.95%)
PPF (360–800)	436.08 ± 33.24	64.30 ± 6.27
Ratio		
Red/Far‐red	3.60	1.02

*Note*: The percentage values in parentheses represent the percentage over photosynthetic photon flux (PPF).

Real‐time PCR analyses on the shade marker genes *YUC9* (*Solyc06g083700*) and PHYTOCHROME INTERACTING FACTOR (*PIF7b*) (*Solyc06g069600*)^17^, ^18^ performed on leaf material from plants grown under HD or LD demonstrated that plants responded to HD at the molecular level. Indeed, in HD plants the up‐regulation of the auxin biosynthetic gene *YUC9* and the down‐regulation of *PIF7b* was observed, indicating that tomato plants responded to the low R/FR signal under HD conditions (Fig. 2(e),(f)). The molecular confirmation of SAS activation through gene expression changes further validated the morphological observations and indicated the involvement of phytochrome‐mediated signaling cascades in the response.^19^

### High density effects biomass and photosynthetic performance of plants subjected to drought

**Figure 3:**
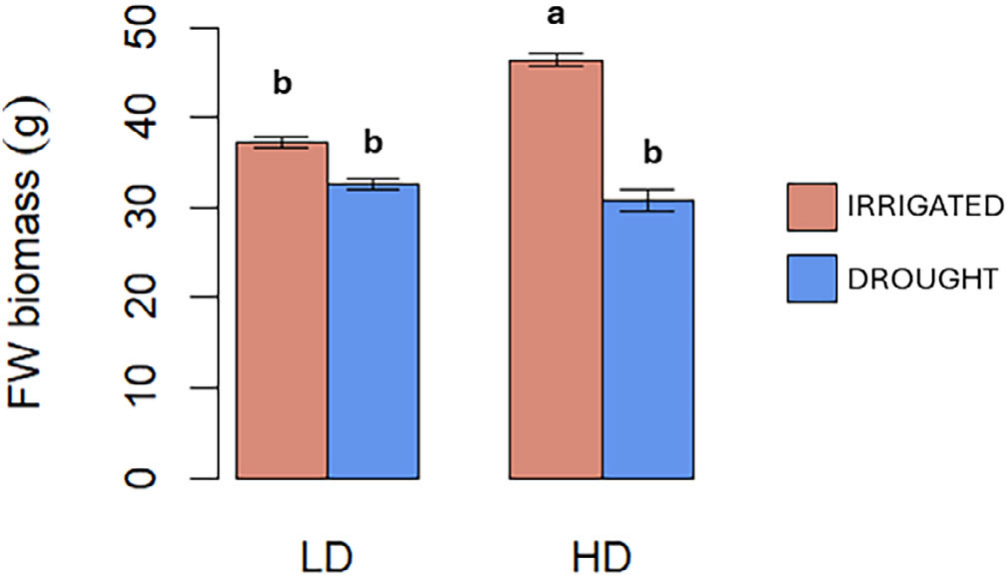
Effect of low density (LD) or high density (HD) without (red chart) or with drought (blue chart) on fresh weight (FW) biomass of tomato plants grown in growth chambers. Letters indicate significant differences according to Tukey *post hoc* test (*P* < 0.05).

**Figure 4:**
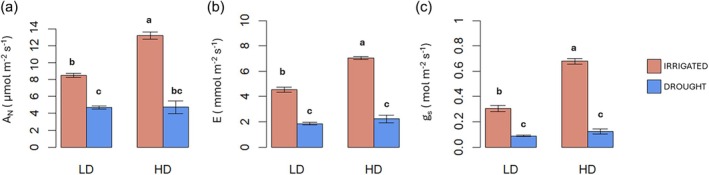
Effect of low density (LD) or high density (HD) without (red chart) or with drought (blue chart) on net photosynthesis (*A*_n_), transpiration rate (*E*) and stomatal conductance (*g*_s_) in tomato plants grown in growth chambers. Letters indicate significant differences according to Tukey *post hoc* test (*P* < 0.05).

Having demonstrated that plants under HD responded to the low R/FR signal at the molecular and morphological level, we next wanted to investigate how this could affect the response of plants to abiotic stress. Therefore, plants under LD or HD were subjected or not to drought by water withholding. The fresh biomass and all the photosynthetic parameters measured were significantly affected by the interaction of the two factors drought and density (D × W) (Table S3). FW biomass analysis revealed that irrigated plants grown under HD attained significantly higher biomass (45.8 g) compared to all other treatments, suggesting that HD conditions can enhance growth under optimal water application (Fig. 3). The remaining treatments (LD irrigated, LD drought, and HD drought) showed statistically similar FWs (37.6, 32.1, and 30.3 g, respectively). DW biomass followed a slightly different pattern, with tomato plants under both HD and drought having reduced dry biomass compared to all other treatments (Table S4), suggesting that HD conditions may exacerbate the effects of drought on biomass accumulation. The higher FW but similar DW between LD and HD irrigated plants might also indicate differences in tissue water content, with HD plants possibly maintaining higher hydration levels. Surprisingly, plants grown under HD with adequate water supply exhibited enhanced photosynthetic rates compared to LD plants with the same water conditions. As shown in Fig. 4(a), highest net photosynthesis (*A*_n_) values were registered in irrigated HD plants (~13 μmol m^−2^ s^−1^), followed by irrigated LD plants (~8.5 μmol m^−2^ s^−1^), while drought significantly reduced rates under both density conditions (~4.5–4.8 μmol m^−2^ s^−1^). Transpiration rate (*E*) and stomatal conductance (*g*_s_) (Fig. 4(b),(c)) followed similar patterns, with irrigated HD plants showing the highest values. The higher photosynthetic rate, together with the higher stomatal conductance and transpiration rate here observed in HD plants was previously observed in other crops under similar conditions.^20^ This counterintuitive enhancement of photosynthesis under HD challenges conventional understanding that crowding inevitably reduces photosynthetic efficiency due to light limitation. The response observed in this study parallels findings by Burbano *et al*.,^7^ who observed enhanced photosynthetic rates when plants were exposed to proximity shade. These physiological adjustments likely represent compensatory mechanisms to maximize light capture efficiency under low light and altered spectral conditions. Consistently, in some species leaves under low light may accumulate more pigments and decrease leaf dry matter per unit area to enhance light capture and harvesting opportunities.^21^, ^22^ Accordingly, HD plants had higher chlorophyll content compared to LD plants, with increases of 32.39% for chlorophyll a and 75.82% for chlorophyll b (Tables S3 and S4). Similar adaptive increases in chlorophyll content have been reported in species under shade conditions.^23^ However, drought generally inhibits chlorophyll synthesis or accelerates its degradation,^24^ reducing the ability of plants to receive and transfer light energy and altering their photosynthetic function. Analogously, chlorophylls a and b contents were significantly reduced by water withholding (Table S4).

### High density affects the oxidative stress and proline accumulation caused by drought

As expected, drought stress increased H_2_O_2_ and MDA levels in both HD and LD plants (Tables S3 and S4). However, it is noteworthy that under HD, there was a decrease in H_2_O_2_ (−22%) compared to LD, in contrast with results obtained by Gao *et al*.^25^ in maize. This reduction in oxidative stress markers under HD conditions suggests that moderate shade might provide protective effects against drought‐induced oxidative damage, possibly through reduced light intensity and consequent lower reactive oxygen species generation.^26^, ^27^

**Figure 5:**
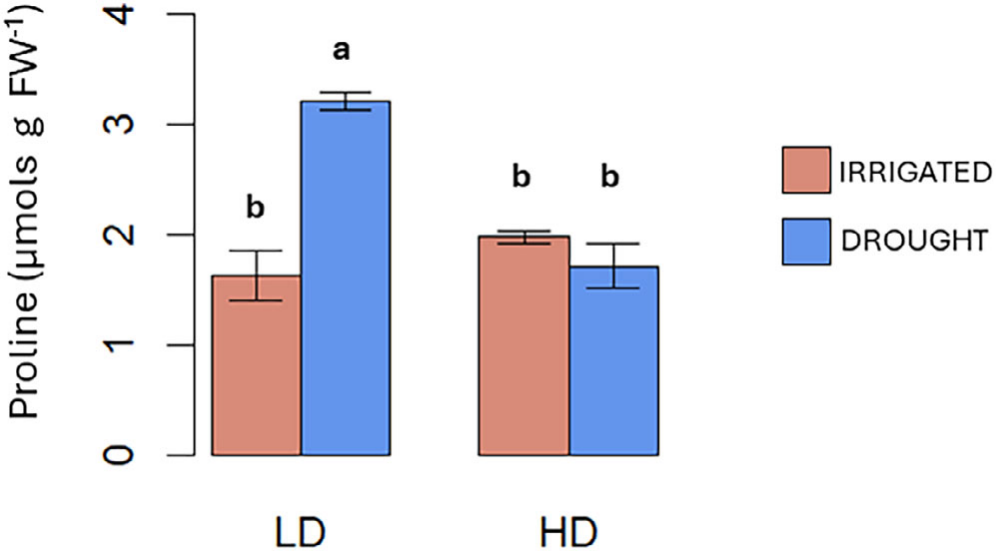
Effect of low density (LD) or high density (HD) without (red chart) or with drought (blue chart) on proline content in tomato plants grown in growth chambers. Letters indicate significant differences according to Tukey *post hoc* test (*P* < 0.05).

**Figure 6:**
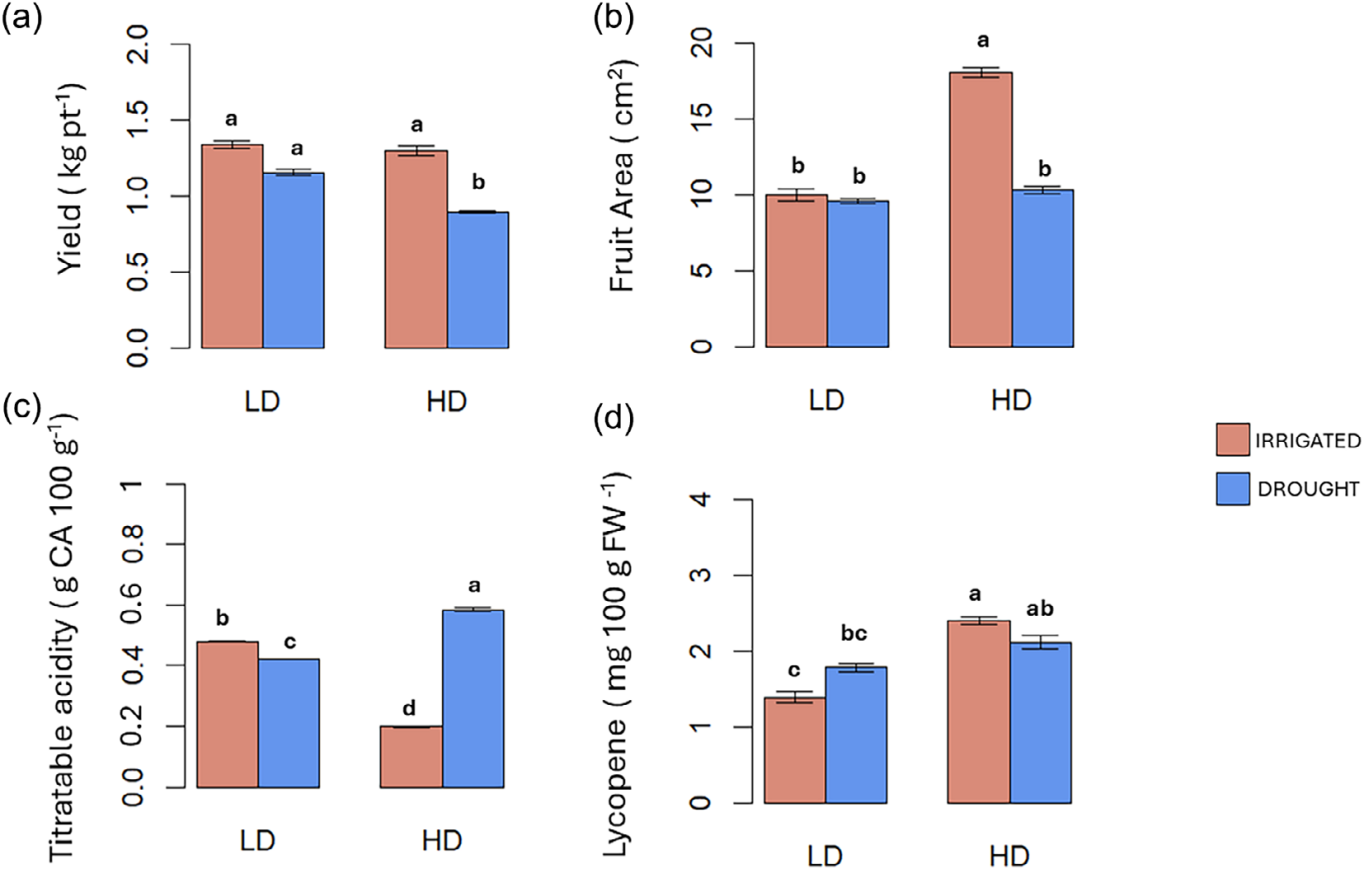
Yield component and physicochemical properties of tomato fruits from plants under low density (LD) or high density (HD) planting without (red chart) or with drought (blue chart) in open field conditions. Letters indicate significant differences according to Tukey *post hoc* test (*P* < 0.05).

As shown in Fig. 5, plants under LD and drought accumulated significantly higher proline levels (3.14 mg g^−1^ FW) compared to all other treatments. This differential response in proline accumulation suggests that plants under HD may employ different physiological strategies to cope with water stress compared to LD plants. This is consistent with the fact that proline synthesis occurs under light,^28^, ^29^ and thus the lower light levels to which HD plants grew may have impaired the synthesis of this osmo‐protectant. Proline synthesis has been identified as an adaptation to improve plant tolerance to osmotic stress (salt, drought).^30^ This adaptation, that is missing in HD, could account for the higher tolerance to drought of LD plants compared to HD, as indicated by the results on fresh and dry biomass (Fig. 3 and Table S4), and further supported by the results on yield (Fig. 6(a)).

### High density and drought affect fruit quality and yield traits in tomato plants grown under low or high density in open field

Next, we investigated the effect of both HD and drought on tomato final yield and fruit quality. In order to get robust data on productivity, tomato plants were grown in open field under HD or LD and subjected or not to drought. Interestingly, high planting density during tomato cultivation increased final yield per hectare compared to low planting density (Table S4). However, HD had no effects on final yields per plant and under HD plants maintained statistically similar yield per plant compared to LD (Fig. 6(a)). Several works on fruit yield and planting density in tomato have been conducted in different locations of the globe evaluating in‐row spacing that went from 15 up to 75 cm; however, different results were obtained suggesting that the effect of in row‐spacing on final yield strongly depend on the area in which the experiments were conducted.^2^ The different fruit yield components were significantly affected by the interaction of the two factors drought and density (D × W) (Table S3). As shown in Fig. 6(a), tomato yield per plant was significantly affected by the combination of HD and drought stress, and combined HD and drought treatment produced substantially lower yields per plant compared to all other treatments.^31^, ^32^

Fruit area measurements (Fig. 6(b)) displayed a different pattern, with HD irrigated plants producing significantly larger fruits (17.8 cm^2^) compared to all other treatments, suggesting a possible compensatory mechanism for reduced fruit number. The physicochemical properties of tomato fruits were also affected by the HD and drought treatments, as shown in Fig. 6. Titratable acidity (Fig. 6(c)) was lower in HD plants but increased significantly under combined HD and drought, reaching 0.50 g TA 100 g^−1^. The observed lower acidity in larger fruits grown under HD conditions could be explained by a possible dilution effect. As the fruit grows, the accumulation of organic acids may not keep pace with the increase in volume, resulting in a lower concentration of acids per unit of tissue. Therefore, the reduced acidity observed in larger fruits may be due to a differential in scale between fruit growth and acid biosynthesis or accumulation.^33^, ^34^ In terms of fruit firmness, no difference was found (Table S4). Most importantly, lycopene content (Fig. 6(d)) increased by 72.75% in fruits from HD plants and did not decrease with drought stress. In line with these results, Song *et al*.^9^ previously demonstrated that low R/FR light irradiation induced the synthesis of lycopene in tomato fruit. An investigation into the carotenoid content of the fruit revealed no statistically significant variations within the β‐carotene composition of the sample (Table S4). This finding has considerable implications for tomato nutritional quality and suggests that manipulation of planting density could be leveraged as a strategy to enhance specific phytonutrient concentrations in fruit crops. Similar enhancement of secondary metabolites under shade conditions has been documented in medicinal plants,^35^ but the application of this principle to improve food nutritional value in major crops represents an innovative approach.

## CONCLUSIONS

**Figure 7:**
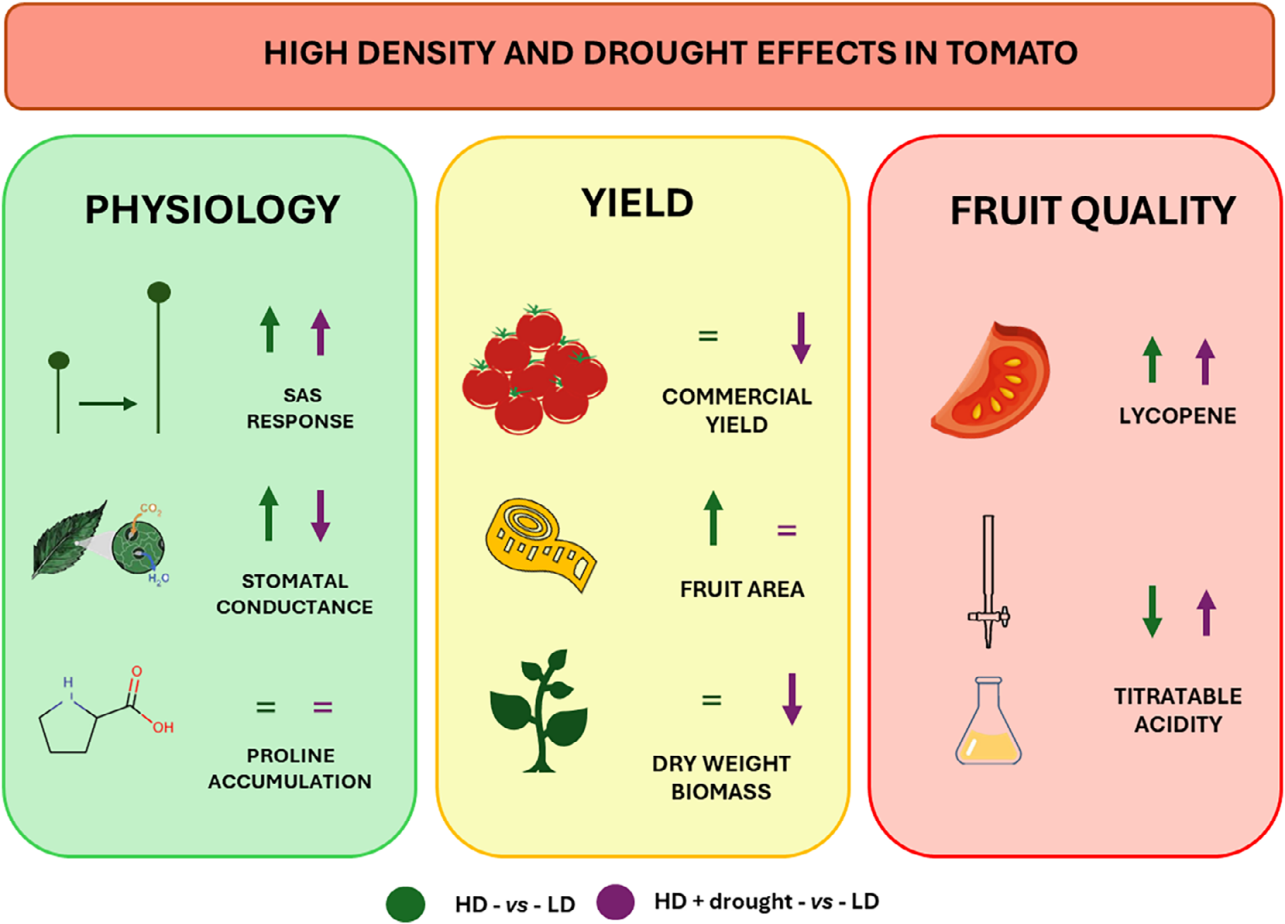
The figure illustrates the main effects of high density (HD) and drought on tomato physiology, yield and fruit quality, highlighting key changes under HD with and without drought compared to low density (LD).

There has been extensive research into increasing planting densities as a strategy to enhance yield per unit of cultivation area.^36^ However, our study addresses critical knowledge gaps regarding the morpho‐physiological effects of combined higher planting density and abiotic stress on tomato plants, and how these interactions influence final fruit yield and quality (Fig. 7). Through extensive experiments in both controlled and open field conditions, we have shown that the relationship between HD cultivation and drought stress is more complex than previously understood. Our findings reveal that HD cultivation triggers classic SAS responses but, contrary to conventional expectations, enhances photosynthetic rates compared to LD plants when water supply is adequate. This unexpected physiological adaptation is accompanied by increased chlorophyll content, particularly chlorophyll b, suggesting a compensatory mechanism to maximize light capture efficiency under reduced light conditions. Furthermore, the modified light environment under HD cultivation (characterized by lower R/FR ratio) led to significant increases in fruit lycopene content, highlighting potential nutritional quality benefits despite lower per‐plant yields. Importantly, our research shows that the combination of HD and drought reduced overall yield per plant more than the single stress, but the improved fruit quality parameters observed suggest that optimized HD management could be a viable strategy for sustainable intensification of tomato production in water‐limited environments. These results challenge simplistic views of combined stress effects and suggest that carefully managed HD planting could be leveraged as a multifunctional approach that balances trade‐offs between yield quantity, resource efficiency, and nutritional quality. Future research should focus on optimizing planting density thresholds for different cultivars and environmental conditions, as well as exploring how these findings might translate to other horticultural crops facing similar resource constraints. The insights gained from this study contribute significantly to developing climate‐resilient cultivation practices that can help address the dual challenges of increasing food production and adapting to water scarcity in the face of climate change.

## CONFLICTS OF INTEREST

The authors have no conflicts of interest to declare.

## Supporting information


**Table S1.** Sequences of adopted primers for amplifying by real‐time qPCR selected shade markers genes.

**Table S2.** The data are mean ± standard deviation of different biological replicates in growth chamber trial in Morocco. Asterisks indicate significant differences among treatments (HD *versus* LD) based on Student's *t*‐test (*P* < 0.05*; *P* < 0.01**; *P* < 0.001***).

**Table S3.** Analysis of variance (ANOVA) for the effects of planting density (D), water regime (W), and their interaction (D × W) on different parameters measured.

**Table S4.** The data are mean ± standard deviation of different biological replicates. Asterisks indicate significant differences among treatments (HD_CTRL *versus* LD_CTRL§ and within each density treatment DROUGHT *versus* CTRL*) based on Student's *t*‐test (*P* < 0.05*; *P* < 0.01**; *P* < 0.001***).

## Data Availability

All data generated or analysed during this study are included in this published article (and its supplementary information files).
