# Supplementary material for: Interactive effects of high planting density and drought on physiological traits and yield in tomato
Source: J Sci Food Agric. 2025 Dec 3;106(5):2648–55. doi: 10.1002/jsfa.70368 (PMC12967680; doi:10.1002/jsfa.70368)
Supplement: Supplementary file 1 — Table S1. Sequences of adopted primers for amplifying by real‐time qPCR selected shade markers genes. [file JSFA-106-2648-s004.docx]

**Table S1.** Sequences of adopted primers for amplifying by Real-Time qPCR selected shade markers genes.

| **PRIMER** | **SEQUENCE** |
| --- | --- |
| *Yuc9*-qF | ACCGTTGAACTTGTCACTGG |
| *Yuc9*-qR | GCACCAGCTAGCCCTTTCC |
| *Pif7b*-qF | GCGCTCCCCTCATTTATCCA |
| *Pif7b*-qR | TTTGGGGCTGATGGATTCGG |
| *Ef 1-α-*qF | AAGCTGCTGAGATGAACAAG |
| *Ef 1-α-*qR | GTCAAACCAGTAGGGCCAAA |
