# Supplementary material for: Interactive effects of high planting density and drought on physiological traits and yield in tomato
Source: J Sci Food Agric. 2025 Dec 3;106(5):2648–55. doi: 10.1002/jsfa.70368 (PMC12967680; doi:10.1002/jsfa.70368)
Supplement: Supplementary file 2 — Table S2. The data are mean ± standard deviation of different biological replicates in growth chamber trial in Morocco. Asterisks indicate significant differences among treatments (HD versus LD) based on Student's t‐test (P < 0.05*; P < 0.01**; P < 0.001***). [file JSFA-106-2648-s003.docx]

**Table S2.** The data are mean ± standard deviation of different biological replicates in growth chamber trial in Morocco. Asterisks indicate significant differences among treatments (HD -*vs*- LD) based on Student’s *t*-test (p<0.05*; p<0.01**; p<0.001***).

|  | **LD** | **HD** | |  |
| --- | --- | --- | --- | --- |
| Plant height | | 56.24±0.80 | | 61.7±1.4^***^ |
| Stem diameter | | 7.40±0.49 | | 7.2±0.6 |
| Internode length | | 29.67±3.08 | | 37.6±6.7^*^ |
| DW_biomass | | 22±1.17 | | 21.2±2.1 |
