# Supplementary material for: Interactive effects of high planting density and drought on physiological traits and yield in tomato
Source: J Sci Food Agric. 2025 Dec 3;106(5):2648–55. doi: 10.1002/jsfa.70368 (PMC12967680; doi:10.1002/jsfa.70368)
Supplement: Supplementary file 3 — Table S3. Analysis of variance (ANOVA) for the effects of planting density (D), water regime (W), and their interaction (D × W) on different parameters measured. [file JSFA-106-2648-s002.docx]

**Table S3.** Analysis of variance (ANOVA) for the effects of planting density (D), water regime (W), and their interaction (D × W) on different parameters measured.

|  | D | W | D*W |
| --- | --- | --- | --- |
| A_N_ | * | *** | * |
| g_s_ | *** | *** | *** |
| E | ** | *** | * |
| Chla | *** | *** | ns |
| Chlb | *** | *** | ns |
| Proline | *** | *** | * |
| Hydrogen peroxide ** | | *** | ns |
| Lipid peroxidation | ns | *** | ns |
| Proline | *** | *** | * |
| FW Biomass | * | *** | ** |
| DW Biomass | ns | ** | ns |
| Yield *per* plant | ** | *** | * |
| Yield *per* hectare | *** | *** | *** |
| Fruit area | *** | *** | *** |
| TA | *** | *** | *** |
| Lycopene | *** | ns | ** |
| β-carotene | * | ns | ns |
| Firmness | ns | ns | ns |
