# Supplementary material for: Interactive effects of high planting density and drought on physiological traits and yield in tomato
Source: J Sci Food Agric. 2025 Dec 3;106(5):2648–55. doi: 10.1002/jsfa.70368 (PMC12967680; doi:10.1002/jsfa.70368)
Supplement: Supplementary file 4 — Table S4. The data are mean ± standard deviation of different biological replicates. Asterisks indicate significant differences among treatments (HD_CTRL versus LD_CTRL§ and within each density treatment DROUGHT versus CTRL*) based on Student's t‐test (P < 0.05*; P < 0.01**; P < 0.001***). [file JSFA-106-2648-s001.docx]

**Table S4.** The data are mean ± standard deviation of different biological replicates. Asterisks indicate significant differences among treatments (HD_CTRL -*vs*- LD_CTRL ^§^ and within each density treatment DROUGHT -*vs*- CTRL ^*^) based on Student’s *t*-test (p<0.05*; p<0.01**; p<0.001***).

|  | **LD_CTRL** | **LD_DROUGTH** | **HD_CTRL** | **HD_DROUGHT** |
| --- | --- | --- | --- | --- |
| ChlA | 142.04±15.23 | 122.15±15.55^**^ | 188.05±11.35^§§§^ | 161.25±10.59^***^ |
| ChlB | 67.02±7.86 | 53.88±3.68^***^ | 117.84±10.53^§§§^ | 96.33±10.38^**^ |
| Hydrogen peroxide | 0.18±0.04 | 0.29±0.03^***^ | 0.13±0.03^§§^ | 0.26±0.07^***^ |
| Lipid peroxidation | 0.02±0.002 | 0.03±0.003^***^ | 0.02±0.002 | 0.03±0.007^***^ |
| DW Biomass | 5.17±0.52 | 4.91±0.44 | 5.23±0.65 | 4.12±0.52^*^ |
| β-carotene | 1.17±0.53 | 0.89±0.33 | 1.23±0.33 | 1.51±0.57 |
| Firmness | 22.4±2.58 | 20±5.16 | 21.4±1.87 | 23±6.74 |
| Yield *per* hectare | 40.45±3.8 | 34.95±2.78^*^ | 80.95±10.21^§§§^ | 55.75±3.12^**^ |
